# Design, Fabrication and Characterization of a MEMS-Based Three-Dimensional Electric Field Sensor with Low Cross-Axis Coupling Interference

**DOI:** 10.3390/s18030870

**Published:** 2018-03-15

**Authors:** Biyun Ling, Chunrong Peng, Ren Ren, Zhaozhi Chu, Zhouwei Zhang, Hucheng Lei, Shanhong Xia

**Affiliations:** 1 State Key Laboratory of Transducer Technology, Institute of Electronics, Chinese Academy of Sciences, Beijing 100190, China; lingbiyun15@mails.ucas.ac.cn (B.L.); crpeng@mail.ie.ac.cn (C.P.); renren@mail.ie.ac.cn (R.R.); czz_casie@163.com (Z.C.); zhangzhouwei15@mails.ucas.ac.cn (Z.Z.); leihucheng16@mails.ucas.ac.cn (H.L.); 2 University of Chinese Academy of Sciences, Beijing 100049, China

**Keywords:** electric field sensor, three-dimensional, cross-axis coupling interference, symmetry

## Abstract

One of the major concerns in the development of three-dimensional (3D) electric field sensors (EFSs) is their susceptibility to cross-axis coupling interference. The output signal for each sensing axis of a 3D EFS is often coupled by electric field components from the two other orthogonal sensing axes. In this paper, a one-dimensional (1D) electric field sensor chip (EFSC) with low cross-axis coupling interference is presented. It is designed to be symmetrical, forming a pair of in-plane symmetrically-located sensing structures. Using a difference circuit, the 1D EFSC is capable of sensing parallel electric fields along symmetrical structures and eliminating cross-axis coupling interference, which is contrast to previously reported 1D EFSCs designed for perpendicular electric field component measurement. Thus, a 3D EFS with low cross-axis coupling interference can be realized using three proposed 1D EFSCs. This 3D EFS has the advantages of low cross-axis coupling interference, small size, and high integration. The testing and calibration systems of the proposed 3D EFS were developed. Experimental results show that in the range of 0–120 kV/m, cross-axis sensitivities are within 5.48%, and the total measurement errors of this 3D EFS are within 6.16%.

## 1. Introduction

Electric field measurement is essential in many scientific and industrial fields, such as power system monitoring [1,2], meteorological phenomena studies [3,4], and electrostatic protection [5,6], etc. Responding to different measurement environments (e.g., temperature and state of matter) and different characteristics of the measured electric field (e.g., frequency, amplitude, duration and direction) in these applications, plenty of principles and techniques have been applied in electric field measurement, and a variety of electric field sensors (EFSs) have been developed. In the last two decades, with the rapid development of Micro-electro-mechanical Systems (MEMS) technology, much literature on electric field sensor chips (EFSCs) has emerged, providing various EFSCs with the advantages of low power cost, small size, high integration, and convenience for batch production. These EFSCs can be classified into three categories according to their working principles, namely, induction charge [7,8,9,10,11,12], electrostatic force [13,14], and steered-electrons [15]. These EFSCs focus on direct current (DC) and low-frequency alternating current (AC) electric field measurement. The most reported EFSCs are single-axis ones, whose sensing axes are perpendicular to their upper surfaces. However, in many cases (e.g., in high voltage infrastructure [1] and atmospheric studies [16,17]) the electric field has an unknown direction before measurement. Thus, sensors that can accurately detect and quantify three-dimensional (3D) electric fields in natural and artificial environments are in demand.

A typical 3D EFS consists of three orthogonal sensing axes to sense three Cartesian components of the electric field simultaneously. One of the major concerns in developing 3D EFS is its susceptibility to cross-axis coupling interference. In other words, the output signal for each sensing axis of the 3D EFS is often coupled with electric field components from the two other orthogonal sensing axes. For example, the output signal for the *X*-axis of a 3D EFS is probably coupled with electric field components from the *Y*- and *Z*-axes, affecting the accuracy of 3D electric field measurement. Its performance index is cross-axis sensitivity expressed as the ratio of the measured sensitivity in the cross-direction to the measured sensitivity in the sensing direction, which is widely employed in evaluating 3D sensors, including 3D accelerometers [18].

Aimed at developing MEMS-based 3D EFSs with low cross-axis coupling interference, some relevant works have been reported concerning algorithms and novel micromachined sensor structures. Wen [19] firstly derived a 3 × 3 coupling sensitivity matrix with pre-measured cross-axis sensitivities, and employed this matrix to compensate the measurement results from three typical one-dimensional (1D) EFSCs. Li [20] introduced a genetic algorithm (GA) to determine the coupling sensitivity matrix. Although their methods can be easily implemented and are minimally restrictive, their studies merely rely on the algorithm rather than sensor structure and are incapable of eliminating cross-axis coupling interference at the source. Higher cross-axis coupling interference means higher cross-axis sensitivity in that matrix, and consequently leads to larger 3D electric field measurement errors. Ling [21] investigated cross-axis coupling interference elimination in terms of symmetrical structures combined with a difference circuit. He firstly presented a single-chip 3D EFSC, where two pairs of sensing elements are located symmetrically to form a cross-like shape with each pair to obtain the *X*- and *Y*-axis electric field components, respectively. Sensing elements in each pair are designed to be identical in structure, so that cross-axis coupling interference on the *X* and *Y* sensing axes can be eliminated theoretically with a difference circuit. However, a symmetrically-located sensing structure is not available for the *Z*-axes sensing element. Moreover, this device suffers from large driving voltage and low sensitivity owing to its great integrity and complicated sensor structure.

For the sake of accurate 3D electric field measurement, a MEMS-based 3D EFS with low cross-axis coupling interference is developed in this paper. The 3D EFS is composed of three identical 1D EFSCs forming three orthogonal sensing axes. In contrast to previously reported 1D EFSCs designed for perpendicular electric field component measurement, the proposed 1D EFSC is designed to be symmetrical, forming a pair of in-plane symmetrically-located sensing structures, which makes the 1D EFSC capable of sensing parallel electric field along symmetrical structures and eliminating cross-axis coupling interference. Moreover, the testing system and calibration system of the proposed 3D EFS are developed.

## 2. Structure Design and Working Principle

**Figure 1:**
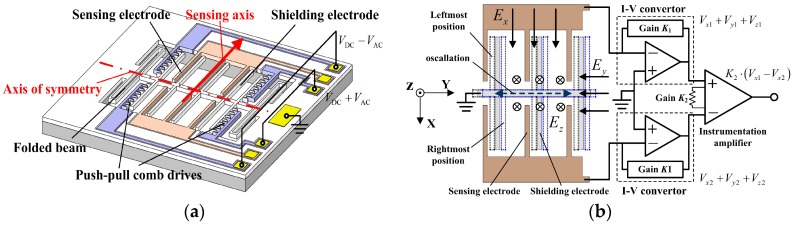
Schematic view of the proposed one-dimensional (1D) electric field sensor chip (EFSC). (**a**) The 1D EFSC is designed to be symmetrical, and its sensing axis is along symmetrical structures. *V*_DC_ is the direct current (DC) bias voltage of driving signal, and *V*_AC_ is alternating current (AC) voltage of driving signal; (**b**) two arrays of fixed sensing electrodes are connected to the difference circuit. *E_x_*, *E_y_*, and *E_z_* are the *X*-, *Y*- and *Z*-axis electric field components, respectively. *V_x_*, *V_y_* and *V_z_* are the outputs of sensing electrodes array with respect to *E_x_*, *E_y_* and *E_z_* after the I-V converter.

The 1D EFSC is designed to be symmetrical, as shown in Figure 1a, and is fabricated on a silicon-on-insulator (SOI) die, as described in Section 4. It is constructed from a resonator that is laterally actuated by push–pull comb drives. Strip-type electrodes are employed as sensing electrodes and shielding electrodes. Two arrays of fixed sensing electrodes are symmetrically placed and interdigitally coupled to their coplanar shielding electrodes. Two identical folded beams are arranged adjacent to push–pull comb drives and connected to shielding electrodes, with other ends anchored to ground, forming a movable shutter. The folded beam works as the elastic structure for the resonator since it can provide a much larger linear deflection range. The 1D EFSC is based on charge induction to sense the external electric field. Yang [11] once presented the operational principle of the lateral EFSC in detail. When the movable shutter is laterally excited by push–pull comb drives, the shielding electrodes oscillate along axis of symmetry, covering the sidewalls of sensing electrodes periodically, as shown in Figure 1b. As a result, the strength of electric field on the strip-type sensing electrodes varies periodically in the external electric field, and the alternating current is consequently induced. Notably, this external electric field can be either perpendicular to the chip or not. Sensing electrodes cause the distortion of the nearby electric field, so that an electric field can be generated on sensing electrodes even with a parallel external electric field. This is explained in detail in [21].

In contrast to typical 1D EFSCs designed for sensing electric field components perpendicular to the chip, the proposed 1D EFSC focuses on sensing parallel electric field along symmetrical structures. The 1D EFSC is assumed to be placed on the *X*-*Y* plane with its sensing axis parallel to the *X*-axis, and its axis of symmetry located on *Y*-*Z* plane and parallel with the *Y*-axis, as shown in Figure 1b. A uniform electric field is applied. For the *X*-axis electric field component, the *Y*-*Z* plane is an equipotential plane as the 1D EFSC is symmetrical in structure. Thus, it can be inferred that the electric flux on the surfaces on these two sensing arrays are opposite theoretically, so that the induced charges on two arrays of sensing electrodes by *X*-axis electric field component are opposite as well. However, the induced charges on two arrays of sensing electrodes by the *Y*- and *Z*-axis electric field components are identical. The outputs of two arrays of electrodes are connected to a difference circuit consisting of two identical I-V convertors and an instrumentation amplifier, which can eliminate the influences of electric field components along *Y*- and *Z*-axes, and leave the *X*-axis electric field component alone. Detailed difference circuit information is presented in [22]. Therefore, it can be inferred that each 1D EFSC is merely sensitive to electric field component along its sensing axis after the difference circuit.

Moreover, for gaining large vibration amplitude with low excitation voltage, each 1D EFSC works at its resonant frequency. A modal simulation of the movable shutter structure was conducted to predict its resonant frequencies and its corresponding vibration modes. Using the finite element analysis (FEA) method, the frequencies of the first five orders resonant modes were calculated to be 2552 Hz, 3496 Hz, 3532 Hz, 4684 Hz, and 7433 Hz respectively, in which the resonant frequency of lateral vibration mode was 2552 Hz. The key parameters of the proposed 1D EFSC are listed in Table 1. The size of 1D EFSC is 6.4 mm × 6.4 mm.

## 3. Modeling of the 1D EFSC

**Figure 2:**
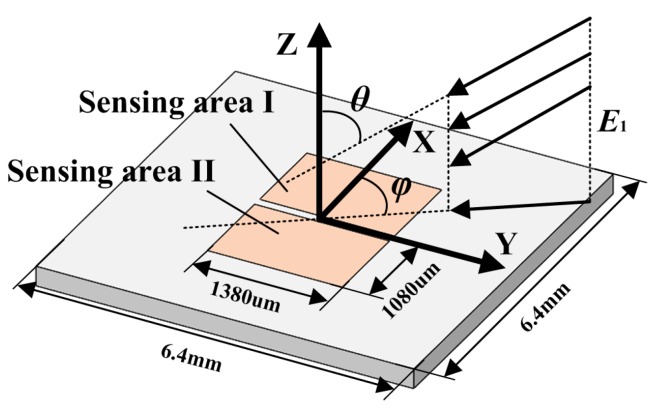
Simulation model for the response of 1D EFSC to a 3D electric field with different directions.

Modeling analysis was performed to validate the capability of the proposed 1D EFSC in sensing parallel electric field along symmetrical structures and eliminating cross-axis coupling interference. The simulation model is illustrated Figure 2, in which the 1D EFSC was simplified based on its outlines. Two arrays of electrodes were simplified into two rectangular sensing areas. The serpentine springs and push–pull comb drives were disregarded. Notably, these two rectangular sensing areas are electrically isolated, but the upper surface of this model is equipotential due to its testing circuit. The 1D EFSC was placed on the *X*-*Y* plane with the *Y*-axis working as its axis of symmetry, so that its sensing axis was along the *X*-axis. A uniform electric field with strength of *E*_1_ was applied. The angle between the electric field and the *Z*-axis was *θ*. The angle between the projection of the electric field on the *X*-*Y* plane and *X*-axis was *φ*. Therefore,

(1) $$E_{x}=-E_{1}\sin\theta \cos\varphi$$

(2) $$E_{y}=-E_{1}\sin\theta \sin\varphi$$

(3) $$E_{z}=-E_{1}\cos\theta$$

where *E_x_*, *E_y_*, and *E_z_* are the *X*-, *Y*-, and *Z*-axis components of the external electric field, respectively. *Q*_A1_ and *Q*_A2_ are the induced charges on sensing area I and sensing area II, respectively.

(4) $$Q_{x}=Q_{A1}-Q_{A2}$$

where *Q_x_* is the differential output of these two sensing areas. Theoretically, the characteristics of *Q_x_* with respect to *θ* and *φ* should be in accordance with *E_x_*, and have no relationship with *E_y_* and *E_z_*.

The FEA method was employed to simulate the induced charge on these two sensing areas with different external electric field directions. The boundary conditions for this simulation strictly follow those of calibration. Firstly, a cubic free space with sufficiently large dimensions was created, in which the simplified 1D EFSC was arranged at its center. Two opposite sides of this cubic free space were selected to apply opposite voltages for generating a uniform electric field. Thus, an electric field from different directions with respect to the 1D EFSC was generated by rotating the rectangular free space around its center, while the simplified 1D EFSC was kept still. *E*_1_ was set to be 10 V/m. *Q_x_* was calculated when the direction of the applied electric field changed circumferentially.

**Figure 3:**
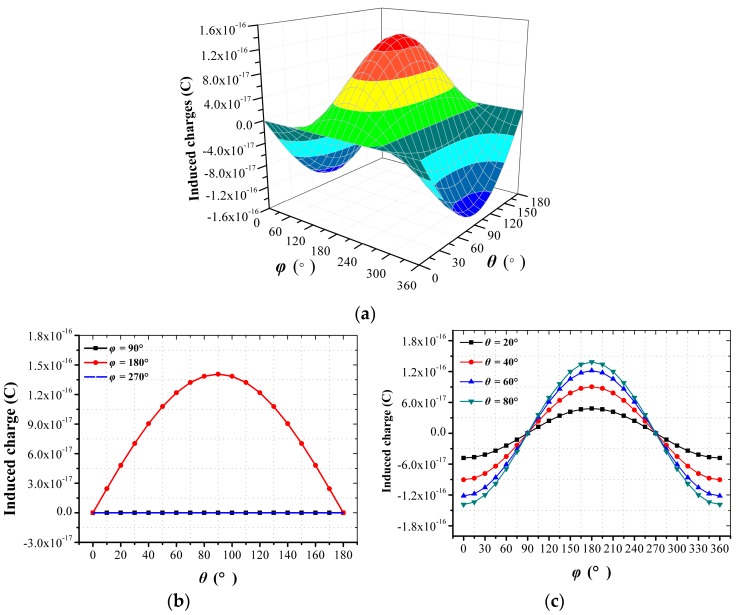
Simulation results of the proposed 1D EFSC’s response to the electric field with different directions. (**a**) *Q_x_* with respect to *θ* and *φ*; (**b**) *Q_x_* with respect to *θ* when *φ* is 90°, 180°, and 270°; (**c**) *Q_x_* with respect to *φ* when *θ* is 20°, 40°, 60°, and 80°.

The relationship between *Q_x_* and different combinations of *θ* and *φ* are presented in Figure 3a. Then, some sections were taken from the 3D plot to illustrate this relationship better. As shown in Figure 3b, when *φ* was 0°, the curves about *Q_x_* versus *θ* showed good sinusoidal characteristics, whereas *Q_x_* was zero when *φ* was 90° and 270°. It can be inferred that *Q_x_* is merely sensitive to *E_x_*, and has no relationship with *E_y_* and *E_z_*. As shown in Figure 3c, when *θ* was 20°, 40°, 60° and 80°, the curves with respect to *Q_x_* versus *φ* showed good sinusoidal characteristics. This illustrates that the characteristics of *Q_x_* with respect to *θ* and *φ* were in good accordance with *E_x_*. The simulation results matched the previous assertion that the characteristics of *Q_x_* with respect to *θ* and *φ* should be in accordance with *E_x_*, and have no relationship with *E_y_* and *E_z_*. Therefore, simulation results prove that the proposed 1D EFSC is capable of measuring electric field component along its sensing axis accurately without cross-axis coupling interference.

## 4. Fabrication of 1D EFSC

**Figure 4:**
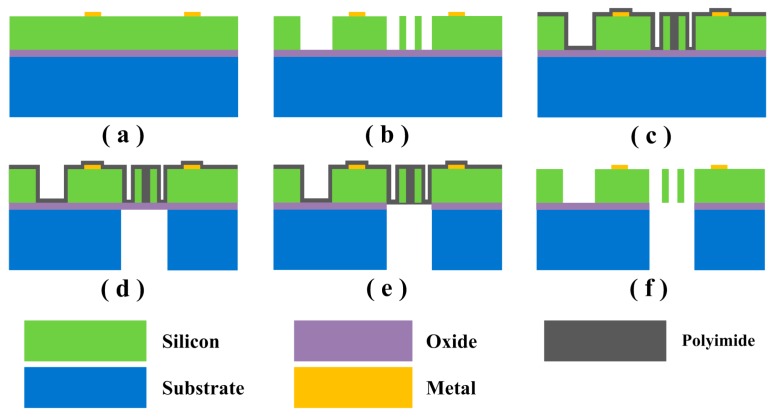
Main steps of the SOI micromachining process.

The proposed 1D EFSC was fabricated on a SOI die. An n-type doped wafer with a 25-μm-thick silicon layer, a 2-μm-thick oxide layer, and a 300-μm-thick substrate layer was chosen. Sensing electrodes, shielding electrodes, push–pull comb drives, and folded beams were formed by etching the silicon layer down to the oxide layer. These structures were all suspended and anchored to the substrate layer. The main steps of fabrication corresponding to Figure 4a–f are described as follows:

(a) Metal pads measuring 50 nm (chrome) and 150 nm (gold) are sputtered on the silicon layer.
(b) Deep reactive ion etching (DRIE) is utilized to etch the structure silicon down to the oxide layer, forming the structures mentioned above.
(c) Polyimide is spin-coated on the patterned structure silicon layer as the front protection material.
(d) DRIE is utilized to etch completely through the substrate layer from the back side, stopping at the oxide layer.
(e) Exposed oxide layer is then removed by CHF_3_ from the back side.
(f) Polyimide is removed using oxygen plasma.

**Figure 5:**
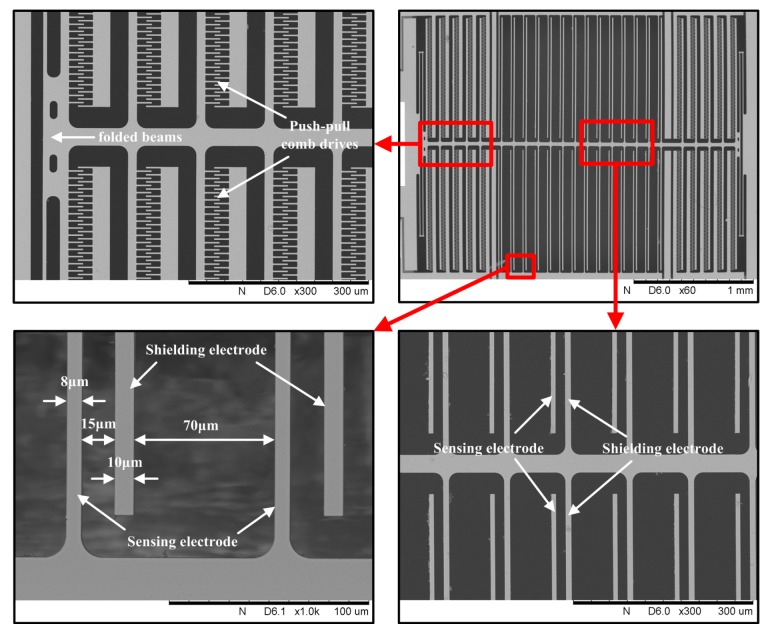
SEM photos of the 1D EFSC. The widths of the sensing and shielding electrodes are 8 μm and 10 μm, respectively; the gaps between sensing electrode and adjacent two shielding electrodes are 15 μm and 70 μm, respectively.

Notably, the backside etching of substrate layer and exposed oxide layer are necessary steps for releasing the movable shutter. The polyimide layer is essential in protecting the structure on silicon layer in backside etching of the substrate layer and exposed oxide layer. The scanning electron micrograph (SEM) photos of the fabricated 1D EFSC are shown in Figure 5.

## 5. Assembly and Modeling of 3D EFS

**Figure 6:**
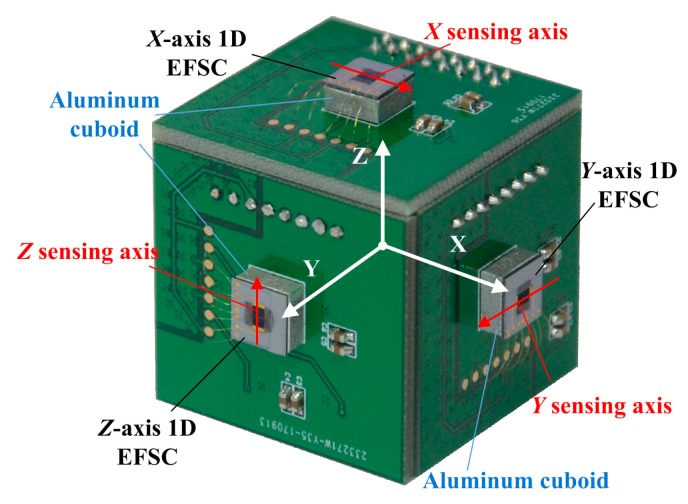
The 3D electric field sensor (EFS) and its local coordinate system. These three printed circuit boards (PCBs) formed three orthogonal facets of a cube. The center of the cube is coincident with the origin of the coordinates. The size of 3D EFS is 3 cm × 3 cm × 3 cm.

Figure 6 illustrates the 3D EFS. Each 1D EFSC is stuck to an aluminum cuboid placed at the center of a printed circuit board (PCB). The cuboid is utilized to magnify the electric field distortion on chip, so as to improve the sensitivity of each 1D EFSC. Each PCB contained two identical I-V converters and an instrumentation amplifier in order to amplify the output of 1D EFSC locally. Its schematic view is shown in Figure 1. By arranging these three PCBs forming three orthogonal sensing axes, a 3D EFS with low cross-axis coupling interference was realized.

**Figure 7:**
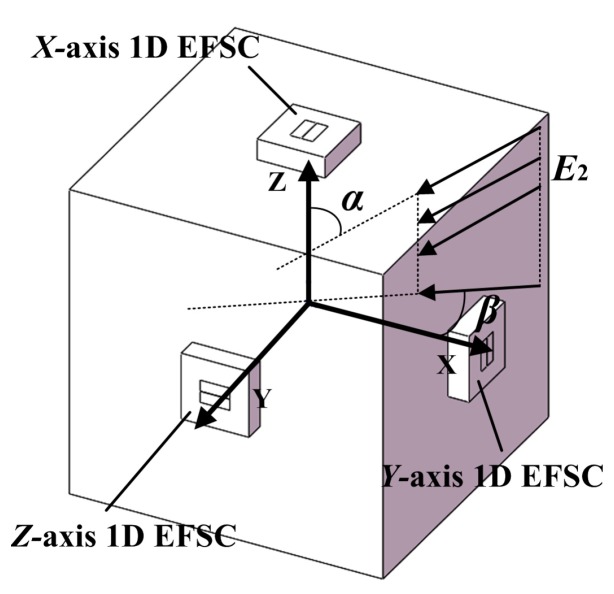
Simulation model for the response of the 3D EFS to a 3D electric field with different directions.

Modeling analysis was performed to investigate the developed 3D EFS’s responses to 3D electric fields. The 3D EFS was simplified based on its outlines as well. The simplified simulation model was a combination of three orthogonally arranged plates, as shown in Figure 7. The 1D EFSC was simplified into two rectangular sensing areas, similar to that of Section 3, and arranged at the center of each plate. Electric field with strength of *E*_2_ was applied. The angle between the electric field and *Z*-axis was *α*. The angle between the projection of the electric field on the *X*-*Y* plane and *X*-axis was *β*. The FEA method was used to calculate the induced charge on each 1D EFSC with respect to *α* and *β*. Notably, the FEA modeling work was similar to that in Section 3.

**Figure 8:**
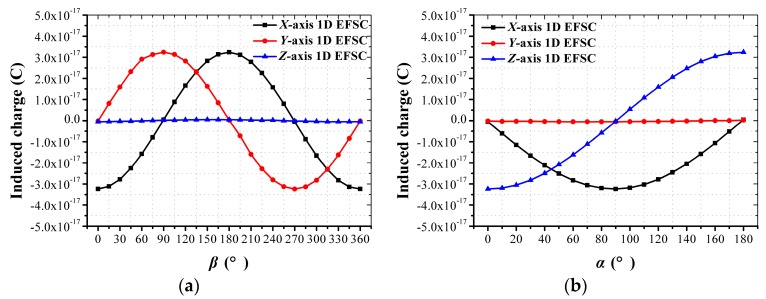
Simulation results of the proposed 3D EFS’s response to the electric field with different directions. (**a**) Induced charge along each sensing axis with respect to *β* when *α* is 90° and *E*_2_ is 10 V/m; (**b**) induced charge along each sensing axis with respect to α when *β* is 0° and *E*_2_ is 10 V/m.

As shown in Figure 8a, when *α* was 90°, the curves with respect to the induced charge on the *X*- and *Y*-axis 1D EFSCs showed good sinusoidal characteristics with a 90° phase difference, whereas the induced charge on *Z*-axis 1D EFSC was almost zero. As shown in Figure 8b, when *β* was zero, the curves with respect to induced charge on the *X*- and *Z*-axis 1D EFSCs showed good sinusoidal characteristics, with a 90° phase difference, whereas induced charge on the *Y*-axis 1D EFSC was almost zero. These curves were basically in good accordance with the electric field component on the *X*-, *Y*- and *Z*-axes, but there exist residual cross-axis sensitivities, especially in Figure 8a. The sensitivity of the *Z*-axis 1D EFSC to the *Y*-axis electric field components is non-zero, and the cross-axis sensitivities were calculated to be within 1.45%. The simulation model is a combination of three plates rather than cuboid, so that dissymmetry is introduced into this model. Ideally, the cross-axis sensitivities are zero if the proposed 3D EFS is cube-shaped. Therefore, simulation results prove that the proposed 3D EFS is capable of eliminating cross-axis coupling interference effectively but its dissymmetry makes it fail to eliminate cross-axis coupling interference thoroughly.

## 6. Characterization

### 6.1. Testing and Calibration Systems

**Figure 9:**
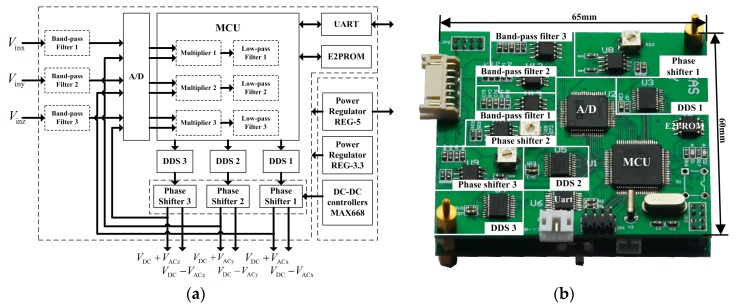
Back-end processing system. (**a**) Schematic view. *V*_DC_ is the 20 V DC bias voltage of the driving signal. *V*_inq_ is the output of the *q*-axis 1D EFSC after the front-end amplification system, *V*_ACq_ is AC voltage of the driving signal of the *q*-axis 1D EFSC, *q* = *x*, *y*, *z*. The microcontroller unit (MCU) connects to three direct digital synthesizers (DDSs) to generate three AC voltages with programmed frequencies. The phase shifter is adopted to provide a 180° phase delay for the AC voltage. The band-pass filter aims to eliminate DC bias and high-frequency noise in the *V*_inq_ so as to increase the signal-to-noise ratio. The back-end processing system interacts with the computer through universal asynchronous receiver/transmitter (UART); (**b**) picture of the back-end processing system. The size of back-end processing system is approximately 65 mm × 60 mm.

To measure the outputs of three 1D EFSCs simultaneously, this paper for the first time developed a miniaturized testing system. Considering that these three 1D EFSCs are independent of each other, the testing system can be divided into three sensing channels. Each sensing channel includes a driving signal generator, I-V converter, instrumentation amplifier, band-pass filter, multiplier, and low-pass filter. Among them, the I-V converter and instrumentation amplifier work as a front-end amplification system, as shown in Figure 1 and Figure 6. The rest form the back-end processing system whose job is to generate driving signals, sample and process the outputs from the front-end amplification system, and upload the measured results. The schematic view is shown in Figure 9.

For each sensing channel, a pair of driving signals, each composed of 20 V DC bias voltage and 1 V AC voltage, are generated to excite the 1D EFSC. Notably, to push–pull the movable structure of the 1D EFSC, these two driving signals are identical but have a 180° phase difference in their AC voltage. Furthermore, to gain a large vibration amplitude, the frequency of AC voltage is close to the resonant frequency of 1D EFSC. The resonant frequencies of these three 1D EFSCs were detected to be 2220 Hz, 2220 Hz, and 2250 Hz, respectively, after the frequency sweep. The simplified structure for modal simulation, error of chip fabrication, and air damping account for the difference between the simulated resonant frequencies and detected resonant frequencies. Multiplier and low-pass filters are created digitally in the MCU. They work as a lock-in amplifier to extract the amplitude of the sensing signal, which is proportional to the applied electric field strength.

**Figure 10:**
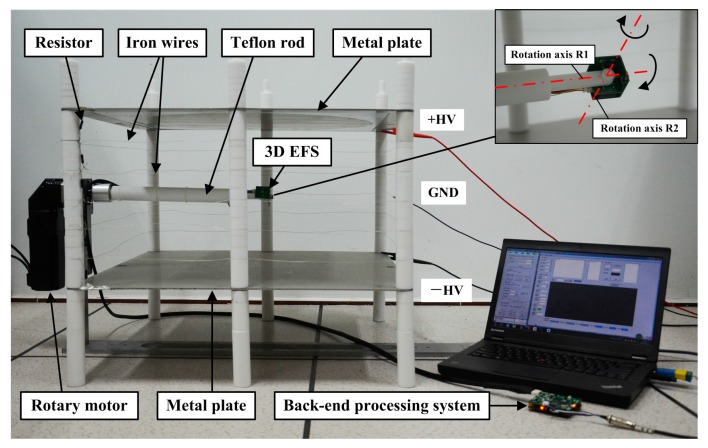
Calibration system. The size of uniform electric field space is 60 cm × 60 cm × 30 cm.

Figure 10 illustrates the calibration system. Two parallel metal plates were applied with opposite voltages respectively to generate a uniform electric field. Equally spaced iron wires, along with equivalent resistances electrically connecting the adjacent two iron wires as well as a metal plate and the adjacent iron wire, were employed to minimize the fringe effect of two parallel metal plates. These two parallel metal plates and iron wires formed a cubic uniform electric field space. The 3D EFS was fixed to one end of a Teflon rod, and arranged at the center of the uniform electric field space. The Teflon rod was parallel with two metal plates. The rod had two orthogonal and intersecting rotation axes, R1 and R2, and its intersection was coincident with the center of the 3D EFS, which caused the center of the 3D EFS to stay still in rotation. Notably, the other end of the rod was connected with a rotary motor, so that the rotation around R1 could be electrically controlled, while the rotation around R2 was manually controlled.

### 6.2. Calibration

**Figure 11:**
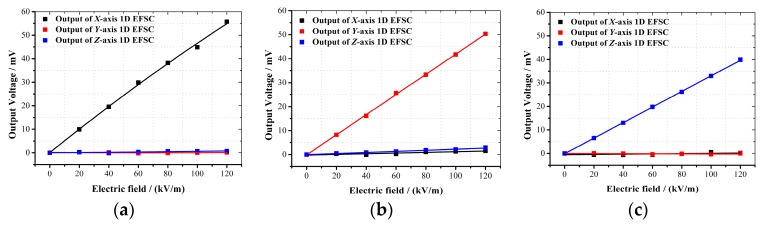
Uniaxial electric field calibration for the 3D EFS. (**a**) The electric field is applied along *X*-axis; (**b**) electric field is applied along *Y*-axis; (**c**) electric field is applied along *Z*-axis.

By rotating the 3D EFS around rotation axes R1 and R2, electric fields parallel with the *X*-, *Y*- and *Z*-axes of the respective 3D EFSs were applied. In each case, the outputs of the *X*-, *Y*- and *Z*-axis 1D EFSCs with respect to the electric field strength ranging from 0 to 120 kV/m were all recorded for calibration, as shown in Figure 11.

The sensitivities of the *X*-, *Y*- and *Z*-axis 1D EFSCs in the three cases are listed in Table 2. It is obvious that the cross-axis sensitivities are within 5.48%. Besides the inherent cross-axis sensitivity described in Section 5, the error induced by assembly and chip fabrication may account for the rest of the cross-axis sensitivity. However, these errors can be estimated and minimized with strict and high standards of chip fabrication and 3D assembly. All the measured linearity errors for these three 1D EFSCs were within 1.8%, which revealed that each 1D EFSC had good linearity.

A comparison of cross-axis sensitivities of the recently reported MEMS-based 3D EFSs was conducted, and the results are listed in Table 3, showing that the cross-axis coupling interference was eliminated effectively in this paper. Furthermore, each 1D EFSC of this 3D EFS was tested in the three roundtrip measurements with the electric field along its sensing axes. In the range of 0–120 kV/m, the uncertainties of these three 1D EFSCs were calculated to be within 1.92%, which revealed that the 3D EFS was insusceptible to noise.

The coupling characteristics can be expressed by the following matrix.

(5) $$\left[\begin{array}{c} V_{x}-V_{x0}\\ V_{y}-V_{y0}\\ V_{z}-V_{z0} \end{array}\right]=\left[\begin{array}{ccc} k_{xx} & k_{xy} & k_{xz}\\ k_{yx} & k_{yy} & k_{yz}\\ k_{zx} & k_{zy} & k_{zz} \end{array}\right]\left[\begin{array}{c} E_{x}\\ E_{y}\\ E_{z} \end{array}\right],$$

where *V_q_* is the output voltage of the *q*-axis 1D EFSC, *V*_q0_ is the zero output voltage of the *q*-axis 1D EFSC, and coupling sensitivity *k*_qi_ is the sensitivity of the *q*-axis 1D EFSC to the electric field in the direction *i*; *i* = *x*, *y*, *z,* and *q* = *x*, *y*, *z.* Therefore, the electric field can be expressed as

(6) $$\left[\begin{array}{c} E_{x}\\ E_{y}\\ E_{z} \end{array}\right]={\left[\begin{array}{ccc} k_{xx} & k_{xy} & k_{xz}\\ k_{yx} & k_{yy} & k_{yz}\\ k_{zx} & k_{zy} & k_{zz} \end{array}\right]}^{-1}\left[\begin{array}{c} V_{x}-V_{x0}\\ V_{y}-V_{y0}\\ V_{z}-V_{z0} \end{array}\right].$$

Thus, in this study, the coupling matrix is given by

$$S={\left[\begin{array}{ccc} k_{xx} & k_{xy} & k_{xz}\\ k_{yx} & k_{yy} & k_{yz}\\ k_{zx} & k_{zy} & k_{zz} \end{array}\right]}^{-1}=\left[\begin{array}{ccc} 2.189 & -0.005 & -0.039\\ -0.071 & 2.380 & -0.164\\ -0.033 & 0 & 3.022 \end{array}\right].$$

### 6.3. 3D Electric Field Measurement and Verification

Experiments were conducted to investigate the measurement accuracy of the developed 3D EFS. The 3D EFS was rotated to several random angles (*θ*_1_, *θ*_2_, *θ*_3_, *θ*_4_, and *θ*_5_). For each angle, electric fields of 50 and 100 kV/m were respectively applied, so that the direction of applied electric field was random. The outputs of the *X*-, *Y*-, and *Z*-axes 1D EFSCs were recorded, and *E_x_*, *E_y_*, and *E_z_* were derived afterwards. The strength of the applied electric field was computed with

(7) $$E=\sqrt{E_{x}^{2}+E_{y}^{2}+E_{z}^{2}}$$

The comparison between the applied and calculated electric fields is listed in Table 4. The measurement errors were of less than 6.16%. Thus, it can be concluded that the calculated electric field of the 3D EFS was basically consistent with the applied electric field, but measurement errors still existed. Systematic errors, assembly-induced errors, and fabrication-induced dissymmetry of 1D EFSC may account for the measurement errors. Likewise, these errors can be minimized with appropriate noise shielding in the testing circuit, and high standards of chip fabrication and 3D assembly.

## 7. Conclusions

In this paper, a novel 3D EFS with low cross-axis coupling interference is presented. The 3D EFS is composed of three identical 1D EFSCs forming three orthogonal sensing axes. Each 1D EFSC is designed to be symmetrical, forming a pair of in-plane symmetrically-located sensing structures. Simulation results proved that the developed 1D EFSC is able to measure parallel electric field along symmetrical structures and eliminate cross-axis coupling interference. The testing and calibration systems of the proposed 3D EFS were developed. The 3D EFS’s response to a 3D electric field with different directions was investigated. Experimental results show that in electric field range of 0–120 kV/m, cross-axis sensitivities are within 5.48%, and the uncertainties are within 1.92%. The total measurement errors of this 3D EFS are within 6.16%.

## Figures and Tables

**Table 1:** The key parameters of the proposed 1D EFSC.

Symbol	Structural Parameters	Value
τ	structure thickness	25 µm
*h*	substrate thickness	300 µm
*w*_sn_	width of sensing electrodes	8 µm
*w*_sh_	width of shielding electrodes	10 µm
*L*_sn_	length of sensing electrodes	1030 µm
*L*_sh_	length of shielding electrodes	1045 µm
*g*	gap between sensing and shielding electrodes in equilibrium position	15 µm
*W*	gap between adjacent two sensing electrodes	95 µm
*N*_e_	number of sensing electrodes	14 × 2
*N*_d_	number of drive combs	84 × 20
*m*_eff_	mass of movable shutter	4.4 × 10^−5^ g
*k*_q_	simulated elastic efficient	11.3 N/m

**Table 2:** Sensitivities of the *X*-, *Y*- and *Z*-axis 1D EFSCs.

Electric Field Direction	*X*-Axis Sensitivity/(mV·kV^−1^·m)	*Y*-Axis Sensitivity/(mV·kV^−1^·m)	*Z*-Axis Sensitivity/(mV·kV^−1^·m)
*X* direction	0.457	0.001	0.006
*Y* direction	0.014	0.420	0.023
*Z* direction	0.005	0	0.331

**Table 3:** Cross-axis sensitivity comparison of the reported MEMS-based 3D EFSs.

MEMS-Based 3D EFS	Year	Cross-Axis Sensitivity
Assembled 3D EFS with three typical 1D EFSCs [20]	2016	≤11.80%
Single-chip 3D EFSC [21]	2017	≤56.62%
3D EFS in this paper	2018	≤5.48%

**Table 4:** Outputs of the 3D EFS and calculated electric fields.

Rotation Angle	Applied Electric Field/(kV·m^−1^)	Output of *X*-Axis 1D EFSC/mV	Output of *Y*-Axis 1D EFSC/mV	Output of *Z*-Axis 1D EFSC/mV	Calculated Electric Field/(kV·m^−1^)	Error
*θ*_1_	50	−1.68	11.06	13.93	48.77	2.46%
	100	−3.42	21.88	29.08	100.40	0.40%
*θ*_2_	50	0.01	18.63	8.41	49.92	0.16%
	100	0.04	37.06	16.77	99.35	0.65%
*θ*_3_	50	6.05	7.22	16.49	53.08	6.16%
	100	11.91	14.10	32.88	105.60	5.60%
*θ*_4_	50	0.01	22.02	−0.01	52.41	4.82%
	100	0.05	43.73	−0.05	104.08	4.08%
*θ*_5_	50	0.01	−11.08	13.88	50.80	1.60%
	100	0.01	−22.00	27.70	101.22	1.22%
